# Effect of Visual Field Test on Intraocular Pressure in Glaucoma Patients

**DOI:** 10.3390/jcm14186356

**Published:** 2025-09-09

**Authors:** Weon Jin Jang, Han Jun Chung, Min Woo Lee, Jung Tae Kim, Hyung-Bin Lim, Kee Sup Park

**Affiliations:** 1 Department of Public Health, Graduate School of Konyang University, Daejeon 35365, Republic of Korea; jwj7677@naver.com; 2 Department of Ophthalmology, Konyang University College of Medicine, Daejeon 35365, Republic of Korea; hanjuncyayo@gmail.com (H.J.C.); bogus1105@gmail.com (M.W.L.); 200679@kyuh.ac.kr (J.T.K.); 3 1.0 Eye Clinic, Daejeon 34946, Republic of Korea; cromfans@hanmail.net; 4 Department of Ophthalmology, Chungnam National University College of Medicine, Daejeon 35015, Republic of Korea; 5 Myunggok Medical Research Institute, Konyang University, Daejeon 35365, Republic of Korea

**Keywords:** glaucoma, intraocular pressure, visual field test

## Abstract

**Objectives:** To evaluate changes in intraocular pressure (IOP) before versus after a visual field test in glaucoma patients. **Methods:** A total of 132 patients with glaucoma and 103 control subjects who visited Konyang University Hospital between August 2024 and May 2025 were included in the study. the right eye of each patient was selected for analysis. Visual field tests were conducted using the Humphrey Visual Field (HVF) analyzer (Zeiss Humphrey, San Leandro, CA, USA) with the SITA standard program (Central 24-2). Intraocular pressure was measured by two ophthalmologists at five time points: before the test and immediately, 10 min, 30 min, and 60 min after the test. **Results:** The average intraocular pressure decreased from 15.09 mmHg before the test to 14.29 mmHg immediately afterward; it declined further to 13.59 mmHg at 10 min in glaucoma patients. It then gradually increased to 15.01 mmHg at 60 min, returning to pre-test levels. Participants were divided into three age groups (40s, 50s, and 60s) for analysis. Across all groups, the IOP followed a similar pattern: a significant decrease for up to 10 min, followed by recovery at 60 min. Although a reduction in IOP was also observed in the control group after visual field testing, the magnitude of the decrease was smaller compared to the glaucoma patients. **Conclusions:** IOP declined immediately after the visual field test and remained lower for up to 10 min. It subsequently returned to baseline by 60 min. Therefore, when measuring the IOP after a visual field test, there is no need to adjust for temporary fluctuations if the measurement is performed 60 min after the test.

## 1. Introduction

Glaucoma comprises a group of progressive optic neuropathies characterized by structural damage to the optic nerve accompanied by corresponding visual field loss. Elevated intraocular pressure (IOP) is the most significant risk factor, and lowering IOP remains the primary therapeutic strategy [1].

IOP is regulated by the continuous flow of aqueous humor and plays a key role in maintaining the eye’s structural integrity and visual function. Normal IOP ranges from 10 to 21 mmHg, with an average of 15 mmHg [2]. Elevated IOP is the strongest risk factor for glaucoma; lowering the IOP is the only proven treatment for slowing or preventing optic nerve damage [3]. Various factors influence IOP, including age, sex, ethnicity, diurnal fluctuations, systemic hemodynamics, exercise, positional changes, refractive errors, and medications [4,5,6,7]. Although a high IOP contributes to glaucoma by damaging the optic nerve, the precise mechanisms underlying IOP elevation remain unclear [8,9,10,11].

The visual field test is a crucial diagnostic tool for assessing glaucoma progression by measuring the extent of visual field defects. It is particularly important in early-stage glaucoma when the visual field may only be partially affected [12,13].

However, there is ongoing debate regarding the relationship between the visual field test and IOP. Previous studies have yielded conflicting results. Bertaud et al. [14] and Adhikari et al. [15] found no significant difference in IOP values before and after the exam, suggesting the minimal physiological influence of the test. In contrast, Lee et al. [16] reported a temporary increase 10 min after the exam, which normalized within 20 min. Similarly, Sawada et al. [17] observed a significant decrease in IOP after the exam. Other studies have reported transient elevations; for instance, Ni et al. [18] found that the IOP increased after testing 22.9% of glaucoma patients, with a more than 20% change in some cases. Li et al. [19] and Recupero et al. [20] also described short-term IOP elevations that returned to baseline within an hour. Moreover, Asrani et al. [21] emphasized that the IOP can fluctuate widely in glaucoma patients, even when the mean values remain within the normal range. Liu et al. [22] further demonstrated that IOP fluctuations were more pronounced in glaucomatous eyes than in healthy eyes, highlighting the importance of understanding dynamic IOP behavior during visual field testing.

Given the lack of conclusive evidence regarding IOP fluctuations and influencing factors, we investigated fluctuations in the IOP in relation to the test and identified specific factors that may drive these changes.

## 2. Methods

### 2.1. Patients

We examined the right eyes of 132 outpatients with open-angle glaucoma and 103 in a control group who visited Konyang University Hospital between August 2024 and May 2025. All glaucoma patients had an open angle confirmed by gonioscopy, glaucomatous optic nerve damage with corresponding visual field defects, and maintained normal IOP while using anti-glaucoma eye drops. The control group consisted of normal patients who visited a glaucoma clinic. Visual field tests meeting reliability criteria (gaze loss ≤ 20%, false-negative responses ≤ 15%, and false-positive responses ≤ 15%) were included in the analysis. Patients with corneal disease, post-cataract surgery status, or post-glaucoma surgery status were excluded. This prospective study was approved by the Institutional Review Board of Konyang University Hospital in the Republic of Korea (IRB number: 2024-05-023-001). It was conducted in accordance with all relevant requirements of the Declaration of Helsinki. Informed consent was acquired from all participants.

### 2.2. Humphrey Visual Field (HVF)

Humphrey Visual Field (HVF) testing (Zeiss Humphrey, San Leandro, CA, USA—SITA standard program, Central 24-2) was used as a quantitative visual field assessment. Patients with dilated pupils were excluded, and myopia was corrected prior to testing.

### 2.3. Intraocular Pressure (IOP)

IOP measurements were obtained from patients attending the glaucoma clinic by two experienced ophthalmologists using the Goldmann applanation tonometer (GAT) (model AT 900, Haag-Streit International, Köniz, Switzerland) at five time points: before the visual field test and immediately, 10 min, 30 min, and 60 min after the test. All measurements were performed under standardized conditions using consistent techniques. Both examiners were glaucoma specialists trained in GAT measurement protocols, and the same equipment was used throughout the study. For staining, 0.5% paracaine (Hanmi Medicine, Seoul, Republic of Korea) and fluorescein strips were applied. For each examination, the average of two consecutive IOP measurements was used for statistical analysis. To assess inter-observer reliability, the intraclass correlation coefficient (ICC) was calculated for baseline IOP measurements. The results demonstrated excellent agreement between the two examiners, with an ICC of 0.982 in the glaucoma group and 0.977 in the control group, indicating high consistency and reliability in the measurement process.

### 2.4. Optical Coherence Tomography (OCT)

An experienced examiner performed OCT measurements using a Cirrus HD OCT 6000 (Carl Zeiss Meditec, Dublin, CA, USA; version 10.0). The 200 × 200 optic disk cube scanning protocol was applied to assess RNFL thickness. We focused on RNFL thickness rather than the ganglion cell layer (GCL) because RNFL is a widely accepted and reproducible marker for detecting early glaucomatous damage. It shows strong correlation with disease progression and is less affected by macular pathology compared to GCL analysis. Moreover, RNFL thinning often appears earlier than ganglion cell loss in the disease course [23].

### 2.5. Statistical Analysis

Statistical analyses were conducted using PASW software, version 27.0 (SPSS Inc., Chicago, IL, USA); demographic characteristics were compared between the two groups using Student’s *t*-test and chi-square test. Changes in IOP were analyzed with repeated-measures analysis of variance along with univariate and multivariate linear mixed models. A *p*-value < 0.05 was considered statistically significant. In the univariate model, age, sex, and a history of diabetes mellitus and hypertension were assessed to determine their influence on changes in IOP. The multivariate model examined variables identified in univariate analysis, while also incorporating additional independent factors to assess their unique effects.

## 3. Results

### 3.1. Demographics

The study included a total of 132 eyes from glaucoma patients and 103 from a control group. The mean age was 58.66 ± 1.36 years for glaucoma patients and 59.26 ± 1.85 years for the control group (*p* = 0.620). There were no significant differences between the two groups in hypertension, DM, sex, axial length, baseline IOP, CCT, BCVA, spherical equivalent, and axial length (all Ps > 0.05; Table 1). Glaucoma patients used an average of 1.52 ± 0.11 anti-glaucoma eye drops. The mean RNFL thickness was thinner in the glaucoma patients than in the control group (80.58 ± 1.41 μm vs. 97.80 ± 7.61 μm, *p* < 0.001).

HVF testing was performed, and the average testing time was significantly longer in glaucoma patients than in the control group (5.71 ± 0.13 min vs. 4.81 ± 0.15 min, *p* < 0.001). The MD measures overall visual field sensitivity loss and is categorized into three stages: early (–2 to –6 dB), moderate (–6 to –12 dB), and advanced (<–12 dB) [24]. Our glaucoma patients had an average MD of –3.02 ± 0.59 dB, consistent with early-stage glaucoma. The pattern for standard deviation was significantly higher in the glaucoma patients compared with the control group (4.56 ± 0.42 dB, 1.98 ± 0.93 dB, and *p* < 0.001). The VFI was lower in glaucoma patients than in the control group (91.88 ± 1.63%. vs. 97.69 ± 2.02%, *p* < 0.001).

### 3.2. Relationship Between IOP Before Versus After the Visual Field Test

**Figure 1:**
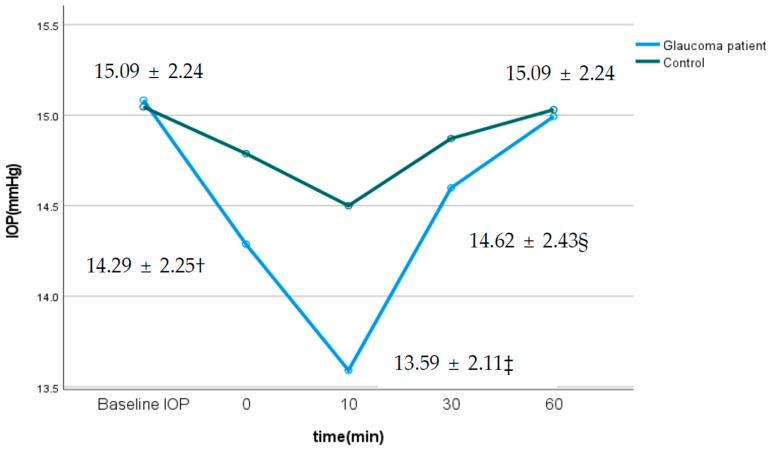
Changes in intraocular pressure in glaucoma patients and the control group over time after visual field test. In glaucoma patients, *p*-value < 0.05, † post hoc test between the baseline IOP and 0 min. ‡ Post hoc test between the baseline IOP and 10 min. § Post hoc test between the baseline IOP and 30 min.

Table 2 and Figure 1 show changes in the IOP according to time point after the exam. The mean IOP was 15.09 ± 2.24 mmHg. Although IOP in the glaucoma patients and control group significantly decreased immediately after the exam, as well as at 10 min and 30 min, it returned to baseline by 60 min. As shown in Figure 1, the difference in IOP before and after the test were statistically significant (*p* < 0.001); the lowest pressure was observed 10 min after the exam. Changes in IOP over time were statistically significant (*p* < 0.001) for both glaucoma patients and the control group. Overall, changes in IOP over time were statistically significant (*p* < 0.001) in both groups. The maximum IOP difference in glaucoma patients and the control group was −1.5 mmHg and −0.53 mmHg, respectively, indicating that the change in IOP was greater in glaucoma patients.

### 3.3. Relationship Between IOP Before Versus After the Visual Field Test, According to Age

**Figure 2:**
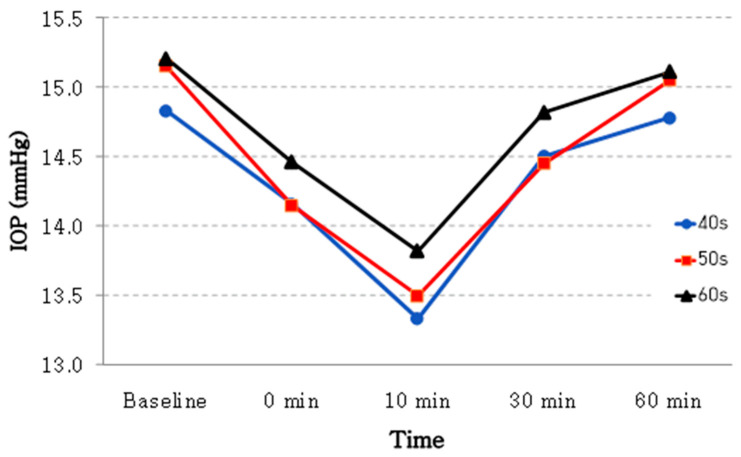
Changes in intraocular pressure over time after visual field test by age group.

A total of 18 eyes from patients in their 40s, 20 eyes from those in their 50s, and 28 eyes from those in their 60s were analyzed. Table 3 and Figure 2 show age-related fluctuations in IOP over time.

In summary, the average IOP of patients in their 60s (15.21 ± 2.54 mmHg) was significantly higher than that of those in their 40s (14.83 ± 2.28 mmHg). Additionally, the decrease in IOP 10 min after the exam was greater among patients in their 40s (–8.98%) than among those in their 60s (–9.08%). Finally, in every age group, the post-exam IOP was significantly lower than the pre-exam value (*p* < 0.001; Table 3).

### 3.4. Factors Influencing Changes in IOP

According to univariate and multivariate linear mixed models, the patient’s age, sex, diabetes, hypertension, use of glaucoma eye drops, average corneal thickness, axial length, spherical equivalent, VFI, MD, and average retinal nerve fiber layer (RNFL) thickness significantly affected changes in IOP (Table 4). In the univariate model, age, hypertension, and average RNFL thickness were significant factors at *p* ≤ 0.1. In the multivariate model, female patients, those with hypertension, and those with thicker RNFLs had lower average IOPs (Table 4).

## 4. Discussion

In glaucoma, elevated IOP can impair ocular blood circulation and cause optic nerve damage, potentially resulting in complete blindness. The global prevalence of glaucoma continues to rise. Previous studies have shown that patients with glaucoma have an im-paired autoregulation of ocular blood flow in response to changes in blood pressure or IOP compared with healthy individuals [25,26]. The measurement of IOP and visual field testing is essential for monitoring disease progression and guiding treatment decisions. Visual field testing is typically performed before the clinical consultation to allow for comparison with previous results.

IOP changes following visual field testing warrant careful consideration. In this study, we measured the IOP at multiple time points (0, 10, 30, and 60 min after the test) and found that it decreased after testing but returned to baseline by 60 min. Table 2 and Figure 1 present the changes in IOP in both glaucoma patients and controls. Both groups demonstrated a transient reduction in the IOP immediately after visual field testing, with statistically significant differences (*p* < 0.001). In the glaucoma group, the maximum reduction occurred at 10 min post-test, with a mean decrease of approximately −1.5 mmHg, followed by a gradual return to near-baseline levels by 60 min. In contrast, the control group exhibited a smaller maximum decrease of about −0.5 mmHg. The more pronounced IOP fluctuation observed in glaucoma patients may reflect impaired ocular blood flow autoregulation. Previous studies have reported that patients with glaucoma show compromised autoregulatory responses to changes in ocular perfusion pressure or IOP [25,26,27]. This reduced capacity for autoregulation may render them more susceptible to physiological stress induced by visual field testing. Although statistically significant IOP changes were also observed in the control group, the absolute magnitude was minimal and recovered rapidly. This suggests that homeostatic mechanisms involving the autonomic nervous system and trabecular meshwork function are better preserved in healthy eyes, resulting in superior IOP recovery compared with glaucoma patients. These findings are relevant not only for the diagnosis and monitoring of glaucoma, but also for patient counseling. Patients can be reassured that post-test IOP fluctuations are transient, decrease within a short period, and eventually return to baseline.

Our findings differ from previous studies that reported increases in IOP after the test. For example, Li et al. [19] found that the IOPs of 31 open-angle glaucoma patients (62 eyes) significantly increased by 12.7% immediately after the test. Similar findings were reported by Recupero et al. [20], who studied 49 primary open-angle glaucoma patients (94 eyes). The mean IOP change was 2.38 ± 3.49 mmHg. However, in both studies, the IOP returned to baseline within 1 h after the exam, as observed in our study. Additionally, Recupero et al. [20] reported higher IOPs in younger patients, which is consistent with our findings.

Our findings are consistent with those of Sawada et al. [17], who also observed a decrease in the IOP after visual field testing in patients with open-angle glaucoma. In their study, the IOP of the right eye decreased from 12.8 ± 2.9 mmHg to 12.3 ± 2.6 mmHg, whereas the left eye remained relatively stable (12.6 ± 2.8 mmHg to 12.5 ± 2.6 mmHg). This difference may be attributable to the right eye being tested first. The reduction in IOP may result from near focusing, which induces ciliary muscle contraction and subsequently enhances aqueous humor outflow [7,24,28]. Similarly, Cassidy et al. [29] reported that in glaucoma patients, engaging in near work for 10 min produced a significant decrease in the IOP compared with looking at a distance for the same duration, a finding in agreement with our results.

As shown in Table 3 and Figure 2, we also found that the IOP was higher in older patients: 14.83 ± 2.28 mmHg among patients in their 40s versus 15.21 ± 2.54 mmHg among those in their 60s. Caprioli et al. [30] reported a relationship between older age and various physiological and structural changes that can increase the IOP. They found that trabecular meshwork dysfunction, changes in lens size, biodynamic alterations in the posterior eye, decreases in retinal ganglion cells, and reduced intracranial pressure were major factors contributing to an elevated IOP. This increase may be due to reduced aqueous humor drainage caused by accommodation dysfunction [20,24]. Psychological stress and nervousness during the test may also contribute due to its demanding nature [31]. The IOP could decrease when the patient relaxes after the exam. Lee et al. [16] reported a significant decrease in IOP 10 min after the exam, followed by a quick recovery within 20 min. This may be attributed to the lower average age of their patients (57.4 ± 11.3 years) relative to ours (58.66 ± 1.36 years).

In this study, we used a univariate model to discover that sex, hypertension, and average RNFL thickness (*p*-value < 0.1)) affect IOP and then used a multivariate model for additional analysis. We found that age, hypertension, and average RNFL thickness were significantly associated with IOP changes (*p*-value < 0.05). Several previous studies have suggested that postmenopausal hormonal therapy in women may contribute to reduced IOP levels [32,33,34]. While our study did not collect specific data regarding hormonal status or therapy, the age distribution of our female participants (predominantly in their 40s to 60s) overlaps with the age group commonly undergoing menopausal transition. Thus, hormonal influences may be one of several factors contributing to IOP differences in this population, though further investigation is required. We also observed that the average IOP of hypertensive patients (14.82 ± 2.04 mmHg) was lower than that of non-hypertensive patients (15.23 ± 2.35 mmHg). This difference may be related to the effects of systemic anti-hypertensive medications, which have been reported to lower ocular perfusion pressure and IOP in some prior studies [26]. However, as detailed information on medication use and blood pressure control was not collected, the interpretation of these findings should be approached with caution. The assessment of RNFL thickness is valuable in evaluating glaucoma progression. A thicker RNFL generally reflects a healthier retina, whereas thinning is an early marker of disease progression [35,36,37]. In our study, patients with a greater average RNFL thickness exhibited less fluctuation in IOP [38].

This study focused on patients with early-stage glaucoma. According to the Early Manifest Glaucoma Trial (EMGT) [39], the risk of glaucoma progression can be reduced by approximately 10% with each 1 mmHg decrease in IOP. In our study, no increase in IOP was observed following visual field testing. These findings may help reassure patients by alleviating concerns about IOP fluctuations after visual field examinations.

This study has several limitations. First, it included only patients with early-stage, open-angle glaucoma; therefore, the findings may not be generalizable to individuals with advanced glaucoma, angle-closure glaucoma, or secondary glaucoma. Second, although we assessed IOP fluctuations after visual field testing, we did not investigate concurrent biometric changes in anterior segment structures, such as anterior chamber depth, lens thickness, or lens position. Third, the follow-up duration was limited to 60 min, which may have missed delayed IOP responses. Fourth, we did not include measurements of the ganglion cell layer (GCL) or assess correlations with other imaging modalities such as optical coherence tomography angiography (OCT-A), which may have provided additional structural insights. Lastly, potential influences such as psychological stress or fatigue during the test—which could affect IOP—were not evaluated.

Despite these limitations, this study has several strengths. Repeated IOP measurements at multiple time points allowed for a robust assessment of temporal changes following visual field testing. Moreover, the inclusion of a control group enabled direct comparisons, highlighting the distinct IOP responses observed in glaucoma patients.

In conclusion, our study indicated that the IOP did not increase before or after visual field testing, eliminating concerns about such effects. Although there was a significant decrease 10 min after the exam, the IOP returned to baseline by 60 min. Therefore, measuring a patient’s IOP 60 min after a visual field test allows for a more accurate assessment. However, patients with mid- to late-stage glaucoma will require further follow-up.

## Figures and Tables

**Table 1:** Demographic characteristics of patients.

	Glaucoma Patients	Control Group	*p*-Value *
Number of patients	132	103	
Age (year, mean ±SD)	58.66 ± 1.36	59.26 ± 1.85	0.432
Sex (male, %)	64 (48.4)	53 (51.5)	0.537 †
Hypertension (%)	19 (14.3)	16 (15.5)	0.795 †
DM (%)	12 (9.1)	11 (10.7)	0.852 †
IOP-lowering medication (*n*)	1.52 ± 0.11	0	0.001
Baseline IOP	15.09 ± 2.24	15.04 ± 2.14	0.844
CCT (mean ± SD)	521.79 ± 5.93	519 ± 4.38	0.580
BCVA (logMAR, mean ± SD)	0.027 ± 0.01	0.023 ± 0.01	0.549
Spherical equivalent (diopter, mean ± SD)	−1.14 ± 0.32	−1.09 ± 0.34	0.534
Axial length (mm, mean ± SD)	23.15 ± 0.11	23.30 ± 0.21	0.155
Mean RNFL thickness (µm, mean ± SD)	80.58 ± 1.41	97.80 ± 7.61	0.001
Humphrey visual field			
Mean deviation (dB)	−3.02 ± 0.59	−0.93 ± 0.36	0.001
Pattern standard deviation (dB)	4.56 ± 0.42	1.98 ± 0.93	0.001
Visual field index (%)	91.88 ± 1.63	97.69 ± 2.02	0.001
VF testing time (min, mean ± SD)	5.71 ± 0.13	4.81 ± 0.15	0.001

* *p*-value from Student’s *t*-test, † *p*-value from the chi-squared test.

**Table 2:** Comparison of IOP variations between before (baseline) and after (0, 10, 30, and 60 min) visual field testing.

	Baseline IOP	0 min	10 min	30 min	60 min	*p*-Value *
Glaucoma patients (*n* = 132)	15.09 ± 2.24	14.29 ± 2.25	13.59 ± 2.11	14.62 ± 2.43	15.01 ± 2.18	**<0.001**
Control group (*n* = 103)	15.04 ± 2.14	14.79 ± 2.06	14.51 ± 2.01	14.85 ± 2.34	15.02 ± 2.19	**<0.001**
						**<0.001**

* *p*-value from the repeated-measures ANOVA (analysis of variation). Significant *p*-values are bolded.

**Table 3:** Comparison of IOP variation between before (baseline) and after (0, 10, 30, and 60 min) visual field testing by ages.

Time		Baseline IOP	0 min	10 min	30 min	60 min	*p*-Value *
**Age groups**	40 s (*n* = 38)	14.83 ± 2.28	14.16 ± 2.19	13.33 ± 2.24	14.50 ± 2.29	14.78 ± 2.28	**<0.001**
	50 s (*n* = 40)	15.15 ± 1.81	14.15 ± 1.95	13.50 ± 1.88	14.45 ± 1.90	15.05 ± 1.85	**<0.001**
	60 s (*n* = 54)	15.21 ± 2.54	14.46 ± 2.49	13.82 ± 2.17	14.82 ± 2.54	15.11 ± 2.36	**<0.001**

* *p*-value from the repeated-measures ANOVA (analysis of variation). Significant *p*-values are bolded.

**Table 4:** Univariate and multivariate linear mixed-effect model determination of factors associated with changes in the IOP.

	Univariate		Multivariate	
Factors	Estimate (95% CI)	*p*-Value	Estimate (95% CI)	*p*-Value
Age	−0.003 (−0.05 to 0.06)	0.119		
Sex (1 = male, 2 = female)	−1.375 (−2.47 to −0.29)	**0.014**	−1.415 (−2.45 to −0.38)	**0.008**
DM	0.114 (−1.63 to 1.86)	0.897		
HTN	−1.079 (−2.26 to 0.10)	**0.073**	−1.231 (−2.34 to −0.12)	**0.030**
IOP-lowering medication	0.281 (−0.37 to 0.93)	0.390		
Central cornea thickness	−0.005 (−0.02 to 0.01)	0.415		
Axial length	−0.333 (−1.07 to 0.40)	0.368		
BCVA(LogMAR)	−4.504 (−12.75 to 3.74)	0.279		
Spherical equivalent	0.069 (−0.16 to 0.29)	0.599		
Visual field index	0.012 (−0.03 to 0.06)	0.583		
Mean deviation	0.071 (−0.05 to 0.19)	0.259		
Pattern standard deviation	−0.107 (−0.28 to 0.07)	0.229		
Average RNFL	−0.046 (−0.09 to 0.003)	**0.069**	−0.057 (−0.10 to −0.01)	**0.017**

IOP = intraocular pressure, BCVA = best corrected visual acuity, logMAR = logarithm of the minimum angle of resolution, RNFL = retinal nerve fiber layer, and Significant *p*-values are bolded (*p* < 0.1).

## Data Availability

The original contributions presented in this study are included in the article. Further inquiries can be directed to the corresponding author.
